# Evaluation of chemical components and quality in Xinhui Chenpi (*Citrus reticulata* ‘Chachi’) with two different storage times by GC–MS and UPLC

**DOI:** 10.1002/fsn3.4154

**Published:** 2024-04-10

**Authors:** Junjie Shi, Lihua Peng, Weixuan Chen, Weilin Qiao, Kui Wang, Yueyang Xu, Jinle Cheng

**Affiliations:** ^1^ Research Center of Chinese Herbal Resource Science and Engineering, School of Pharmaceutical Sciences Guangzhou University of Chinese Medicine Guangzhou Guangdong China; ^2^ Key Laboratory of Chinese Medicinal Resource from Lingnan (Guangzhou University of Chinese Medicine) Ministry of Education of the People’s Republic of China Guangzhou Guangdong China; ^3^ National Enterprise Technology Center, National and Local Joint Engineering Research Center of Ultrafine Granular Powder of Herbal Medicine Zhongshan Zhongzhi Pharmaceutical Group Co. Ltd. Zhongshan Guangdong China

**Keywords:** antioxidant activity, *Citrus reticulata* ‘Chachi’, flavonoids, quantitative detection, volatile compounds

## Abstract

Xinhui Chenpi (XHCP) is a well‐known type of Chenpi (CP) widely used as both a Chinese herb and a food ingredient. While previous studies have explored how the quality of CP changes over time, there has been limited research specifically on XHCP. This study aims to assess the chemical components and quality of XHCP based on total flavonoid content (TF), antioxidant activity (AA), and color value (CV) at two stages: freshly harvested (XHCP‐0Y) and after 3 years of storage (XHCP‐3Y). Thirty‐eight common volatile compounds were identified, and the content of 17 compounds among them, nine nonvolatile compounds, which included one alkaloid (synephrine), three phenolic acids (PA, protocatechuic acid, vanillic acid, and ferulic acid), and five flavonoids (narirutin, hesperidin, sinensetin, nobiletin, and tangeretin), were firstly detected by the newly developed gas chromatograph–mass spectrometer (GC–MS) and ultra‐performance liquid chromatography (UPLC) methods. Compared to XHCP‐0Y, the content of 17 volatile compounds and synephrine decreased in XHCP‐3Y to varying degrees, while the content of PA, five flavonoids, TF, AA, and CV increased. The reduction of dryness caused by volatile compounds and the enhancement of efficacy related to PA, flavonoids, and AA suggested improved quality of XHCP after 3 years of storage. The methods developed in this study show promise for evaluating the quality of XHCP during the aging process.

## Graphical abstract

Two simple GC–MS and UPLC methods were first developed to accurately determine 17 volatile and nine nonvolatile compounds in XHCP. The content of 26 compounds and total flavonoids, along with the color values and the antioxidant activity were utilized to assess the quality of XHCP that was newly harvested and stored for 3 years. The decrease in the levels of volatile compounds and synephrine and the increase in the levels of total flavonoids, phenolic acids, flavonoids, and antioxidant activity during storage would provide evidence supporting the use of XHCP after the aging process.

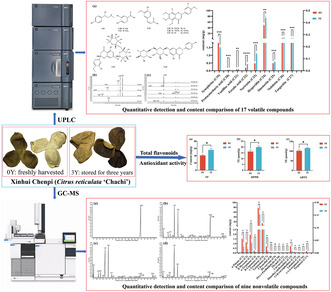

## 1 INTRODUCTION

Citri Reticulatae Pericarpium (CRP), derived from pericarp of *Citrus reticulata* Blanco or cultivars, is widely used in China. Depending on the producing area and source plant, it is classified into Chenpi (CP) and Guangchenpi (GCP), with the latter primarily originating from Guangdong and Guangxi provinces, China. Xinhui Chenpi (XHCP), a well‐known type of GCP, specifically refers to the pericarp of *Citrus reticulata* ‘Chachi’, grown and aged in Xinhui District, Jiangmen, Guangdong, China (Zhang et al., 2020). CRP is frequently utilized in food production for its unique aroma and beneficial properties, commonly found in meat, chicken, tea, and candy. In traditional Chinese medicine, CRP is recognized for its ability to regulate qi, strengthen the spleen, eliminate dampness, and dissolve phlegm (Luo et al., 2018). Various studies have highlighted the diverse positive bioactivities of CRP, such as antioxidant, anti‐inflammatory, antiasthmatic, antimutagenic, antithrombotic, antibacterial, and anticancer effects (Fu et al., 2019; Gosslau et al., 2014; Song et al., 2020; Wang et al., 2017; Zacarias‐Garcia et al., 2021).

The main active components of CRP are flavonoids, volatile oils, and phenolic acids (Chen et al., 2020; Denkova‐Kostova et al., 2021; Gonzalez‐Mas et al., 2019; Luo et al., 2019; Zheng et al., 2020). The *Variorum of the Classic of Materia Medica* (*běn cǎo jīng jí zhù*), an ancient medical text, noted that ‘the older, the better’, implying that the therapeutic effect of CRP is linked to its storage year. Research has extensively explored the changes in flavonoids and phenolic acids during the aging process of CRP due to their significant bioactivity and easy measurability (Li et al., 2021; Luo et al., 2019; Wang et al., 2018; Yang et al., 2021). Phenolic acids and flavonoids like hesperidin, nobiletin, and tangerine have been identified as key components that increase CP during aging, leading to enhanced antioxidant activity (Bian et al., 2022). However, there is limited research specifically on phenolic acids and flavonoids in XHCP. Research on volatile oils, another key active component in CRP, has predominantly focused on their chemical composition, along with qualitative and comparative analysis across different sources and harvest times (Duan et al., 2016; Yi et al., 2015; Zheng et al., 2018). However, there is limited investigation into quantitatively detecting volatile compounds in XHCP and understanding how their variety changes during storage. Ancient texts like Master Lei's Explanation of the Properties of Processed Medicinals suggest that aging process reduces the dryness associated with volatile compounds, a concept supported by modern studies on traditional Chinese medicine. The book also recommends that CRP stored for 2–3 years is more suitable for medicinal use. The Standard for Traditional Chinese Medicine in Guangdong Province now indicates that XHCP can be used after being stored at room temperature for over 3 years. Therefore, XHCP aged for more than 3 years could be utilized in clinical settings, proprietary Chinese medicines, or health products due to its increased effectiveness and reduced dryness. It is important to consider that the cost of XHCP aged over 3 years is notably higher than freshly harvested XHCP (Lan et al., 2020; Liu et al., 2022), and further research is needed to determine if the quality improves during the 3‐year storage period.

Above all, the storage time of 3 years is the key to determining whether XHCP could be used for medicinal purposes. Therefore, this study aimed to more comprehensively compare the changes in the chemical components and quality of XHCP that was freshly harvested (XHCP‐0Y) or stored for 3 years (XHCP‐3Y) to determine its potential medicinal benefits. The analysis included volatile compounds like monoterpene, sesquiterpene, and diterpene, as well as nonvolatile compounds such as flavonoids, phenolic acids, and alkaloids. Gas Chromatography–Mass Spectrometry (GC–MS) was used to identify common volatile compounds and differentiate them using multivariable statistics. Additionally, a detection method combining GC–MS with dMRM mode was first developed and validated for 17 volatile compounds, as well as a UPLC method for nine nonvolatile compounds, including one alkaloid (synephrine), three phenolic acids (PA, protocatechuic acid, vanillic acid, and ferulic acid), and five flavonoids (narirutin, hesperidin, sinensetin, nobiletin, and tangeretin). The study also evaluated total flavonoids (TF), antioxidant activity (AA), and color value (CV) of XHCP‐0Y and XHCP‐3Y, as these parameters are known to indicate quality improvements of CP during storage. The findings suggest that the quality of XHCP may indeed improve after storage, and the detection methods developed in the study hold promise for evaluating XHCP quality.

## 2 MATERIALS AND METHODS

### 2.1 Materials and reagents

HPLC‐grade acetonitrile, methanol, and formic acid were purchased from Merck & Co. (Germany). n‐Hexane (for GC) was purchased from Shanghai Macklin Biochemical Co., Ltd (Shanghai, China). Analytical reagent (AR) methanol was purchased from Guangzhou Chemical Reagent Factory (Guangzhou, China). Eighteen chemical reference substances (CRS) for GC–MS, including α‐pinene (NO.: **C1**, Lot#: P110875, purity ≥99%), β‐pinene (**C2**, P108923, ≥99.0%), β‐myrcene (**C3**, B21632, ≥95%), α‐terpinene (**C4**, B25358, ≥94%), D‐limonene (**C5**, L128108, ≥99%), γ‐terpinene (**C6**, G156812, ≥95%), terpinolene (**C7**, T435004, ≥90%), linalool (**C8**, L106905, ≥98%), citronellal (**C9**, C118628, ≥98%), (+)‐4‐terpineol (**C10**, T162413, ≥93%), terpineol (**C11**, B50740, ≥96%), DL‐carvone (**C12**, C117707, 99%), (S)‐(−)‐perillaldehyde (**C13**, B21699, ≥98%), thymol (**C14**, T118449, ≥99.9%), carvacrol (**C15**, C107162, 99%), dimethyl anthranilate (**C16**, D101434, 98%), (−)‐β‐caryophyllene (**C17**, T334030, ≥98%), and Tridecane (**C18**, T108180, ≥99.5%), and nine CRS for UPLC, including synephrine (**C19**, 110727–202110, 99.8%), protocatechuic acid (**C20**, 110809–201906, 97.7%), vanillic acid (**C21**, 110776–201503, 99.8%), ferulic acid (**C22**, 110773–201915, 99.4%), narirutin (**C23**, B21634, ≥98%), hesperidin (**C24**, 110721–202019, 95.3%), sinensetin (**C25**, B21246, ≥98%), nobiletin (**C26**, 112055–202102, 99.7%), and tangeretin (**C27**, 112054–202102, 99.7%), were purchased from Aladdin Ltd. (Shanghai, China) or Shanghai Yuanye Bio‐Technology Co., Ltd (Shanghai, China), the National Institutes for Food and Drug Control (Beijing, China), or Shanghai Yuanye Bio‐Technology Co., Ltd (Shanghai, China). For antioxidant activity detection, 2,2‐Diphenyl‐1‐picrylhydrazyl (DPPH), 2,2′‐Azinobis‐(3‐ethylbenzthiazoline‐6‐sulphonate) (ABTS), and 6‐Hydroxy‐2,5,7,8‐tetramethylchroman‐2‐carboxylic acid (Trolox) were obtained from Aladdin Ltd. (Shanghai, China).

### 2.2 Preparation of materials

Six batches of XHCP (S1–S6) were obtained from the same plantation in Xinhui District, Jiangmen, Guangdong, China. Samples S1–S3 were freshly harvested in 2022 (labeled as XHCP‐0Y‐1, XHCP‐0Y‐2, XHCP‐0Y‐3) which had not been aged and stored at −20°C prior to the commencement of this study, while samples S4–S6 were harvested in 2019 and had been stored for 3 years in the production areas in Sanjiang town, Xinhui District (labeled as XHCP‐3Y‐1, XHCP‐3Y‐2, XHCP‐3Y‐3). These samples were identified as XHCP by Chinese pharmacist Shiqing Jia and the thin‐layer chromatography (TLC) method for GCP exclusive identification listed in Chinese Pharmacopeia 2020 Edition. All samples were stored at −20°C and ground to a 24‐mesh size before being utilized. Retained samples were stored in the sample room of the National Enterprise Technology Center at Zhongshan Zhongzhi Pharmaceutical Group Co. Ltd, Zhongshan, China.

### 2.3 GC–MS analysis method

#### 2.3.1 GC–MS condition for qualitative and quantitative detection

The MS/MS acquisition was conducted using an Agilent 7890 GC coupled with a triple quadrupole mass spectrometer (QQQ) equipped with an electron ionization (EI) source (7000D). The equipment was fitted with two HP‐5MS UI capillary columns (15 m × 0.25 × 0.25) (Agilent Technologies, Santa Clara, USA) and helium was used as the carrier gas at a constant flow rate of 1.0 and 1.2 mL/min. In the splitless mode, sample solution and mixed CRS solution of 1 μL were injected. The GC oven program consisted of the following steps: the initial column oven temperature was set at 40°C for 2 min. Subsequently, it was increased to 105°C at a rate of 4°C/min, followed by a further increase to 160°C at a rate of 8°C/min and held for 1 min. Finally, it was raised to 280°C at a rate of 20°C/min, resulting in a total run time of 37 min. The injection temperature was set at 280°C, while the ion source temperature and the interface temperature were set at 230°C and 280°C, respectively. For the qualitative identification of volatile compounds in XHCP, we employed full scan mode (MS1 Scan) in MS with a scan range of m/z from 50.0 to 500.0. The quantitative analysis of **C1**–**C17** was conducted under similar conditions as the qualitative analysis. However, we established and utilized the dynamic multiple reaction monitoring (dMRM) mode with one target and multiple qualifier ions.

#### 2.3.2 CRS and sample preparation for GC–MS

To prepare the CRS solution for GC–MS analysis, specific amounts of each of the 17 Chemical Reference Standards (**C1–C17**) were weighed and dissolved in n‐hexane in amber volumetric flasks. This resulted in a solution with a concentration ranging from 1.03 to 25.08 mg per milliliter. Additionally, tridecane (C18) was dissolved separately in n‐hexane at a concentration of 2.87 mg per mL to serve as the internal standard (ISTD). For the sample solution, 100 mg of the sample powder was weighed into a stoppered conical flask. Subsequently, 2 mL of the tridecane solution and 8 mL of n‐hexane were accurately added to the flask. The solution was then ultrasonicated for 15 min at a power of 280 W and a frequency of 42 kHz. After cooling to room temperature, n‐hexane was added to compensate for any lost weight. The mixture was thoroughly shaken and filtered, and the resulting filtrate was used as the sample solution for GC–MS analysis.

### 2.4 UPLC analysis method

#### 2.4.1 UPLC condition

UPLC analysis was performed using the UPLC H‐Class system (Waters, U.S.A.) equipped with a Waters HHS T3 C18 column (100 × 2.1 mm, 1.8 μm) at a flow rate of 0.4 mL/min at 30°C. The detection wavelengths used were 254 nm for **C20** and **C21**, 283 nm for **C19, C23,** and **C24**, and 330 nm for **C22**, **C25**, **C26**, and **C27**. The gradient program for the mobile phases consisted of 0.1% formic acid (phase A) and acetonitrile (phase B) was set as follows: 0–25 min, 1%–50% B; 25–28 min, 50%–70% B; 28–35 min, 70%–100% B; 35–37 min, 100%–1% B; 37–40 min, 1%–1%. The injection volume was 2 μL.

#### 2.4.2 CRS and sample preparation for UPLC

To prepare the CRS solution, individual quantities of eight Chemical Reference Standrads (**C19**–**C23, C25**–**C27**) were accurately weighed and dissolved in methanol to create a mixture containing 36.22, 3.82, 13.45, 0.48, 47.33, 40.7, 74.46, and 70.62 μg of each per milliliter. Additionally, a separate quantity of hesperidin CRS (**C24**) was accurately weighed and dissolved to achieve a concentration of 853.2 μg per milliliter, taking into account its high concentration requirement. For the sample solution, 200 mg of the sample powder was precisely weighed in a stoppered conical flask, followed by the addition of 10 mL of methanol. The sample solution was then ultrasonicated (at a power of 560 W and frequency of 40 kHz) for 30 min. After cooling to room temperature, methanol was added to make up for any lost weight. Subsequently, the mixture was thoroughly shaken and filtered, and the resulting filtrate was utilized as the sample solution for UPLC detection.

### 2.5 GC–MS and UPLC method validation

To validate the two newly developed quantitative methods, several criteria were manipulated, including specificity, linearity, limit of detection (LOD), limit of quantification (LOQ), precision, stability, and recovery. The specificity of the method was determined by testing a blank and a reference solution. The linearity of the method was evaluated by plotting the peak area(s) against the various concentrations of each compound. The minimum concentrations that produced signals at least three times greater than the noise signal (S/N ≥ 3) or 10 times greater (S/N ≥ 10) were used to calculate the LODs and LOQs of the 26 test compounds. To confirm the precision of the method, standards and samples were continuously analyzed six times (*n* = 6). Stability was assessed by analyzing sample solutions at different time points (0, 2, 4, 8, 12, and 24 h). Recovery was determined using the standard addition method.

### 2.6 Color value (CV) detection

A CM‐5 Spectrophotometer (Konica Minolta (China) Investment Ltd., China) equipped with black and white tile standards used to calibrate the instrument before operation was applied to determine the color values of the sample powder, and mainly measures the CIE parameters L*, a*, and b*. The sample powder was mixed with three peels and measured three times to obtain representative data. The average of the three measured data was taken as the final values. The total color difference (∆E*) was calculated using the following equation:

$$∆E^{*}=\sqrt{{\left(a_{3}^{*}-a_{0}^{*}\right)}^{2}+{\left(b_{3}^{*}-b_{0}^{*}\right)}^{2}+{\left(L_{3}^{*}-L_{0}^{*}\right)}^{2}}$$

where $a_{3}^{*}$, $b_{3}^{*}$, and $L_{3}^{*}$ refers to the values of XHCP‐3Y, and $a_{0}^{*}$, $b_{0}^{*}$, and $L_{0}^{*}$ refers to the values of XHCP‐0Y.

### 2.7 Quantification of total flavonoids (TF)

The concentration of TF was determined using a modified colorimetric assay (Ak et al., 2020). Initially, 50 mg of the powder was accurately weighed in a stopper conical flask, followed by the addition of 5 mL of methanol. The solution underwent ultrasonication at a power of 560 W and frequency of 40 kHz for 30 min. After cooling to room temperature, additional methanol was added to compensate for any lost weight. The mixture was then thoroughly shaken and filtered, and 0.1 mL of the successive filtrate was transferred to a 5‐mL volumetric flask, which was then filled with methanol to reach the desired volume. The resulting mixture was once again thoroughly shaken and filtered, and the filtrate obtained was used as the sample solution. A reference solution series was prepared by diluting the stock solution of hesperidin CRS. The concentration of these solutions was ranged from 2.13 to 25.58 μg per milliliter. The absorbance of both the sample solutions and reference solutions was measured against a blank solution at 285 nm using a UV‐2600 Ultraviolet spectrophotometer (Shimadzu (Shanghai) Global Laboratory Consumables Co., Ltd, Shanghai). The concentration of TF in the XHCP samples was calculated using the standard curve, which was established by relating the concentration and absorbance of the reference solution.

### 2.8 Detection of antioxidant activity (AA)

The antioxidant activity of XHCP was determined using the DPPH and ABTS methods, with Trolox used as a CRS. For the DPPH method, a modified version of the method (Karagecili et al., 2023) was followed. 0.4 mL of diluted sample solution was added to a screw cap tube containing 0.4 mL of DPPH solution (64 μM in methanol). The absorbance of the solution was measured at 517 nm after 30 min of incubation in the dark at room temperature. The ABTS method (Uysal et al., 2019) was slightly modified, where 0.15 mL of sample solution and 0.15 mL of ABTS working solution were mixed, and the absorbance of the mixed solution was measured at 734 nm after 6 min of incubation in the dark at room temperature. To evaluate the Trolox equivalent antioxidant capacity (TEAC) of XHCP, calibration curves were prepared using multiple concentrations of Trolox CRS solutions.

### 2.9 Data processing

The identification of 17 volatile compounds (**C1**–**C17**) in sample solutions was achieved by comparing their retention indices (RIs) and mass fragmented patterns with those of CRS or with mass spectra in the NIST20 library. The nine nonvolatile compounds (**C19**–**C27**) were identified by comparing their retention time (RT) and full wavelength absorption chromatogram with those of CRS. Agilent Mass Hunter Quantitative Analysis 10.2 software (QQQ Quantitative Analysis) was used to construct the calibration curves and calculate the content of the 17 volatile compounds. Hierarchical clustering analysis (HCA), principal component analysis (PCA), orthogonal partial least squares discrimination analysis (OPLS‐DA), and variable importance for projection (VIP) were performed using SIMCA‐P software (version 13.0, Umetrics AB, Germany) to assess the relationships of XHCP‐0Y and XHCP‐3Y samples. The original chromatograms were processed and optimized using Adobe Illustrator software (version 2020, Adobe Systems Incorporated, CA, USA). The comparisons and statistical analysis of TF, AA, and content of compounds were performed using GraphPad Prism software (version 9.5, GraphPad Software, USA). Statistical significance was defined as *p* values less than 0.05.

## 3 RESULTS AND DISCUSSION

### 3.1 Analysis and detection of volatile compounds by GC–MS

**FIGURE 1:**
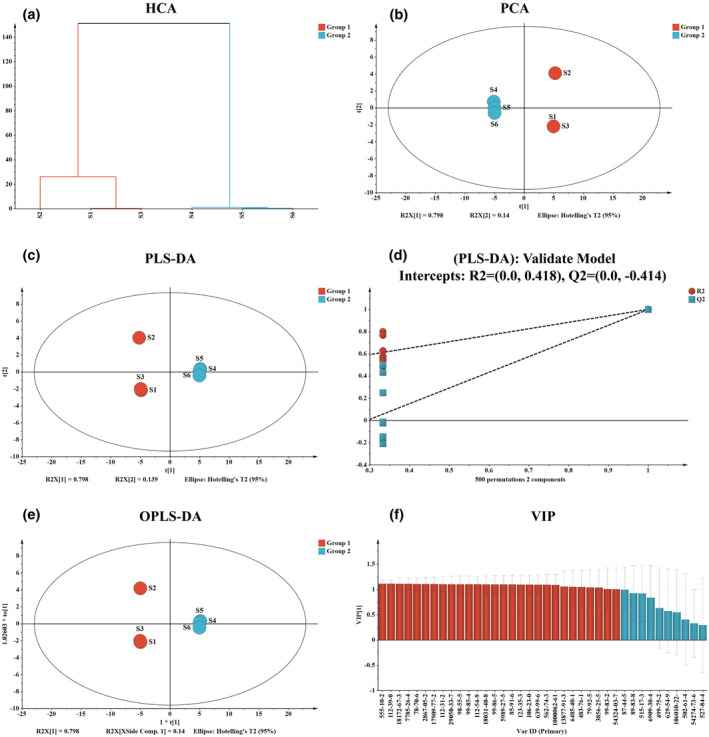
Multivariate analysis of GC–MS data of XHCP with different storage years [S1–S3: XHCP‐0Y (newly harvested) triplicate samples; S4–S6: XHCP‐3Y (stored for 3 years) triplicate samples]. Hierarchical clustering analysis (HCA) (a); Score plots of principle component analysis (PCA) (b); Partial least squares data analysis (PLS‐DA) (c); Permutation test result of PLS‐DA (d); Orthogonal partial least squares data analysis (OPLS‐DA) (e); Plot of the variable importance for the projection (VIP) (f).

The extraction of volatile compounds can be accomplished using various methods, including water vapor distillation, solvent extraction, and scraping (or cold pressed) extraction. Among these techniques, solvent extraction is regarded as the most convenient and quickest, while also yielding a greater number of compounds compared to mechanical oil extraction (Brown et al., 2019). Therefore, considering the polarity of volatile compounds reported in CRP, n‐hexane was chosen as the solvent for extracting XHCP due to its ability to extract a larger number and content of volatile compounds. The prepared sample and CRS solutions were analyzed using the preferred GC–MS condition, resulting in the identification of 38 common compounds in both XHCP‐0Y and XHCP‐3Y samples. Table 1 presents the compound name, retention time (RT), retention index (RI), formula, molecular weight, mass of characteristic ion fragments, and CAS number of these compounds. To examine the distinction between common compounds in the XHCP‐0Y and XHCP‐3Y groups, a combination of unsupervised and supervised classification methods was utilized. The peak areas of the common compounds in two groups were analyzed using SMICA‐P software for statistical analysis. HCA results (Figure 1a **)** segregated samples into two groups based on storage years (S1 ~ S3: XHCP‐0Y and S4 ~ S6: XHCP‐3Y). PCA score chart (Figure 1b **)** also displayed a clear differentiation between the two groups, with principal components *R* ^2^ *X* [1] and *R* ^2^ *X* [2] contributing 79.8% and 14%, respectively. Subsequent establishment of PLS‐DA and OPLS‐DA models (Figure 1c,e) further explored the relationship between the groups, validated by 500 displacement tests (*R* ^2^ *X* = 0.937, *R* ^2^ *Y* = 1.000, Q^2^ = 0.998) as shown in Figure 1d. Analysis of variable importance in projection (VIP) values revealed α‐elemol, α‐terpinene, (S)‐(−)‐perillaldehyde, citronellal, terpinolene, β‐pinene, α‐pinene, and other compounds as key differentiators between two groups (Figure 1f, Table 1).

**TABLE 1:** The common compounds identified by GC–MS with MS1 Scan mode in both XHCP‐0Y and XHCP‐3Y samples.

RT/min	Compound name	RI	Formula	Molecular weight	Mass of characteristic ion fragments	CAS#	VIP
8.89	*α*‐Thujene	911	C_10_H_16_	136.23	93, 91, 77, 92, 79	2867‐05‐2	1.16
9.1	*α*‐Pinene^a^	917	C_10_H_16_	136.23	93, 92, 91, 77, 79	7785‐26‐4	1.14
9.6	Camphene	933	C_10_H_16_	136.23	93, 121, 79, 91, 39	79‐92‐5	1.11
10.51	*β*‐Phellandrene	996	C_10_H_16_	136.23	93, 77, 91, 79, 136	555‐10‐2	1.13
10.59	*β*‐Pinene^a^	964	C_10_H_16_	136.23	93, 41, 69, 79, 77	18,172‐67‐3	1.16
11.21	*β*‐Myrcene^a^	981	C_10_H_16_	136.23	41, 93, 69, 39, 27	123‐35‐3	1.07
11.63	*α*‐Phellandrene	1026	C_10_H_16_	136.23	93, 91, 77, 92, 136	99‐83‐2	1.11
12.09	*α*‐Terpinene^a^	1017	C_10_H_16_	136.23	121, 93, 136, 91, 77	99‐86‐5	1.21
12.4	*o*‐Cymene	1021	C_10_H_14_	134.22	119, 91, 134, 117, 77	527‐84‐4	0.66
12.8	*D*‐Limonene^a^	1030	C_10_H_16_	136.23	68, 93, 67, 136, 121	5989‐27‐5	1.01
13.35	Ocimene^b^	1018	C_10_H_16_	136.23	93, 91, 79, 77, 80	13,877‐91‐3	1.06
13.76	*γ*‐Terpinene^a^	1064	C_10_H_16_	136.23	93, 91, 136, 121, 77	99‐85‐4	1.17
14.74	Terpinolene^a^	1097	C_10_H_16_	136.23	93, 121, 136, 121, 77	586‐62‐9	1.18
15.2	Linalool^a^	1104	C_10_H_18_O	154.25	71, 93, 55, 43, 41	78‐70‐6	1.06
16.55	*(+)‐(E)*‐Limonene oxide	1130	C_10_H_16_O	152.23	94, 108, 43, 67, 93	6909‐30‐4	0.77
17.15	Citronellal^a^	1158	C_10_H_18_O	154.25	41, 69, 55, 95, 43	106‐23‐0	1.19
17.97	*(+)‐4*‐Terpineol^a^	1187	C_10_H_18_O	154.25	71, 111, 43, 93, 86	20,126‐76‐5	1.09
18.45	Terpineol^a^	1190	C_10_H_18_O	154.25	59, 93, 121, 136, 67	98‐55‐5	1.11
19.06	Decanal	1200	C_10_H_20_O	156.26	43, 41, 55, 57, 44	112‐31‐2	1.1
20.65	*DL*‐Carvone^a^	1231	C_10_H_14_O	150.21	82, 54, 39, 93, 108	6485‐40‐1	1.06
22.15	*(S)‐(−)*‐Perillaldehyde^a^	1243	C_10_H_14_O	150.21	68, 67, 79, 107, 39	18,031‐40‐8	1.22
23.29	Thymol^a^	1297	C_10_H_14_O	150.21	150, 135, 91, 136, 115	89‐83‐8	0.92
23.81	Carvacrol^a^	1317	C_10_H_14_O	150.21	150, 135, 91, 136, 77	499‐75‐2	0.64
26.69	Copaene^b^	1376	C_15_H_24_	204.35	161, 119, 105, 93, 41	3856‐25‐5	1.18
27.16	Bicyclosesquiphellandrene	1398	C_15_H_24_	204.35	—	54,324‐03‐7	1.18
27.65	Dimethyl anthranilate^a^	1402	C_9_H_11_NO_2_	165.18	165, 119, 105, 103, 132	85‐91‐6	1.12
27.73	Dodecanal	1412	C_12_H_24_O	184.31	57, 41, 55, 43, 82	112‐54‐9	1.05
27.97	*(−)‐β*‐Caryophyllene^a^	1416	C_15_H_24_	204.35	93, 133, 91, 41, 79	87‐44‐5	1.07
28.84	*α*‐Caryophyllene	1452	C_15_H_24_	204.35	93, 80, 121, 41, 92	6753‐98‐6	0.56
29.5	*(+)*‐epi‐Bicyclosesquiphellandrene	1498	C_15_H_24_	204.35	—	54,274‐73‐6	0.37
29.81	Selina‐4(15),7(11)‐diene	1500	C_15_H_24_	204.35	189, 133, 204, 161, 105	515‐17‐3	0.81
30.09	*α*‐Farnesene	1505	C_15_H_24_	204.35	41, 93, 69, 55, 107	502‐61‐4	0.74
30.4	Naphthalene, 1,2,3,5,6,8a‐hexahydro‐4,7‐dimethyl‐1‐(1‐methylethyl)‐, (1S‐cis)‐	1519	C_15_H_24_	204.35	161, 134, 119, 105, 204	483‐76‐1	1.09
30.84	*α*‐Elemol	1531	C_15_H_26_O	222.36	59, 93, 81, 41, 161	639‐99‐6	1.25
33.06	*α*‐Sinensal	1752	C_15_H_22_O	218.33	93, 55, 134, 79, 41	17,909‐77‐2	1.11
34.2	Hexadecanoic acid, methyl ester	1928	C_17_H_34_O_2_	270.45	74, 87, 43, 55, 143	112‐39‐0	1.12
35.5	Palmitoleamide	2160	C_16_H_31_NO	253.42	—	106,010‐22‐4	0.78
35.58	Hexadecanamide	2182	C_16_H_33_NO	255.43	59, 72, 43, 41, 255	629‐54‐9	0.67

^a^ The compound was identified by authentic standards.

^b^ The isomer was not identified.

**FIGURE 2:**
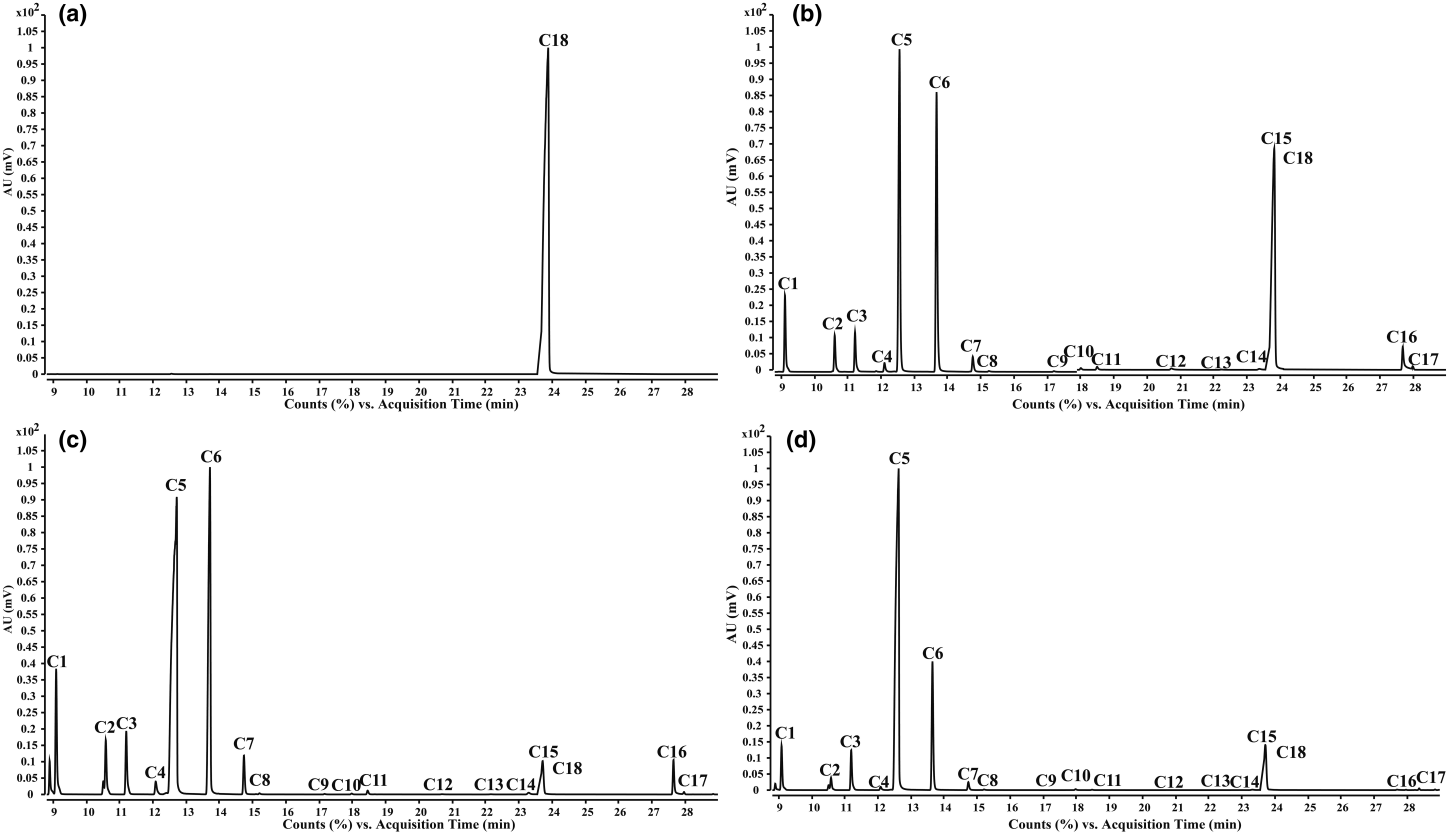
GC–MS detection chromatograms in dMRM mode of blank solution with internal standard (ISTD) (a), 18 chemical reference substances (CRS) solution (b), XHCP‐0Y sample solution (c), and XHCP‐3Y sample solution (d). The compounds **C1**–**C18** are α‐pinene (**C1**), β‐pinene (**C2**), β‐myrcene (**C3**), α‐terpinene (**C4**), D‐limonene (**C5**), γ‐terpinene (**C6**), terpinolene (**C7**), linalool (**C8**), citronellal (**C9**), (+)‐4‐terpineol (**C10**), terpineol (**C11**), DL‐carvone (**C12**), (S)‐(−)‐perillaldehyde (**C13**), thymol (**C14**), carvacrol (**C15**), dimethyl anthranilate (**C16**), (−)‐β‐caryophyllene (**C17**), tridecane (**C18, ISTD**), respectively.

To further investigate changes in compound content in XHCP‐0Y and XHCP‐3Y samples, a quantitative detection method using dMRM mode was first developed. Due to the unavailability of CRS, only 17 out of the 38 compounds listed in Table 1 could be detected (marked with ^a^). The study confirmed optimal sample extraction conditions by comparing compound content and peak areas obtained through different extraction methods, mass concentrations, and extraction times. The results of the optimized extraction conditions are presented in Figure S1. Selection of characteristic ions (m/z) and collision energy was based on multiple trials considering responsiveness, signal‐to‐noise ratio, and linearity as selection criteria. The process and parameters for selecting D‐limonene are illustrated in Figure S2 as an example. Detection chromatograms of the ISTD with blank solution (Figure 2a), 18 CRS (Figure 2b), XHCP‐0Y sample (Figure 2c), and XHCP‐3Y sample (Figure 2d) showed excellent specificity and resolution for these 17 compounds. Method validation, as per Section 2.5, summarized in Table 2, revealed an R squared (*R* ^2^) of the linear equation greater than 0.990, indicating a strong correlation. The relative standard deviation (RSD%) was less than 3%, demonstrating high precision. Both repeatability and stability RSD were less than 5%, further affirming method reliability. LOD ranged from 0.73 to 11.31 ng/mL, while LOQ was between 2.43 and 73.95 ng/mL. Method accuracy ranged from 90% to 110% with a low RSD below 8%, indicating precision. These results clearly show that the developed method is precise, accurate, stable, and suitable for detecting these volatile compounds (Table 3).

**TABLE 2:** Selected characteristic ion (m/z), collision energy (CE), limit of detection (LOD), limit of quantification (LOQ), calibration parameters (linear range and calibration curve), results of method verification (accuracy, precision, repeatability, and stability) for the 17 volatile compounds under study in GC–MS with dMRM mode, under optimized experimental condition.

Compound name	Linear range (μg/mL)	LOD (ng/mL)	LOQ (ng/mL)	Characteristic ions (m/z)	CE (V)	Calibration curve	*R* ^2^	Accuracy (%)	Accuracy (RSD%)	Precision (RSD %)	Repeatability (RSD%)	Stability (RSD%)
*α*‐Pinene **(C1)**	0.73–58.29	0.73	2.43	136, 93^a^, 91, 77	15	*Y* = 10.1457*x* + 0.1735	0.995	97.97	4.48	1.71	2.78	3.35
*β*‐Pinene **(C2)**	0.60–48.16	1.51	5.02	136, 121, 93^a^, 77	13	*Y* = 9.6540*x* + 0.0904	0.998	103.13	5.68	1.34	2.09	4.10
*β*‐Myrcene **(C3)**	0.93–74.69	4.67	15.56	136, 121, 93^a^, 77	13	*Y* = 7.3111*x* + 0.1247	0.997	104.30	6.59	1.86	3.34	3.75
*α*‐Terpinene **(C4)**	0.14–11.49	5.39	17.95	136, 121, 93^a^, 77	15	*Y* = 6.7555*x* − 0.0024	0.996	102.72	4.01	1.73	3.44	4.42
*D*‐Limonene **(C5)**	17.55–1404.28	5.27	17.55	136, 121, 93^a^, 77	15	*Y* = 1.8918*x* + 1.7993	0.992	108.98	7.31	1.59	4.44	3.47
*γ*‐Terpinene **(C6)**	5.03–403.02	7.2	23.98	136, 121, 105, 93^a^, 77	13	*Y* = 5.0583x + 1.1167	0.994	109.56	3.56	1.68	4.53	3.78
Terpinolene **(C7)**	0.29–23.28	3.5	11.64	136, 121, 93^a^, 77	13	*Y* = 5.5674*x* + 0.0172	0.998	98.76	4.69	1.78	4.90	2.15
Linalool **(C8)**	0.08–6.45	12.11	40.35	136, 121, 93^a^, 77	15	*Y* = 4.6227*x* − 0.0031	0.999	99.77	4.16	2.54	1.38	3.62
Citronellal **(C9)**	0.14–11.83	22.2	73.95	154, 139, 121, 93, 69^a^, 41	7	*Y* = 2.3526*x* − 0.0052	0.998	105.74	4.45	2.58	1.57	4.80
*(+)*‐4‐Terpineol **(C10)**	0.09–7.07	8.85	29.47	154, 111, 93, 71^a^, 43	7	*Y* = 3.7792*x* − 0.0017	0.999	102.42	5.15	1.56	2.06	4.21
Terpineol **(C11)**	0.14–11.04	5.92	19.73	136, 121, 105, 93^a^, 77	15	*Y* = 4.6286*x* − 0.0028	0.999	97.19	7.23	2.35	2.00	1.69
*DL*‐carvone **(C12)**	0.07–6.02	11.31	37.65	150, 135, 108^a^, 93, 82, 77, 54	9	*Y* = 6.5836*x* − 0.0061	0.999	97.18	5.26	2.33	2.36	3.42
*(S)‐(−)*‐Perillaldehyde **(C13)**	0.09–7.76	10.78	32.33	150, 135, 122, 107^a^, 91, 79	9	*Y* = 1.2707*x* − 0.0025	0.999	92.33	6.08	1.18	1.74	4.04
Thymol **(C14)**	0.05–4.14	6.22	20.72	150, 136, 135^a^, 92, 91	21	*Y* = 15.2741*x* − 0.0186	0.998	96.03	5.68	3.02	2.82	2.78
Carvacrol **(C15)**	0.16–12.84	8.03	26.75	150, 136, 135^a^, 92, 91	19	*Y* = 11.7207*x* − 0.0414	0.999	100.98	3.38	1.16	2.66	1.92
Dimethyl anthranilate **(C16)**	0.67–54.18	10.17	33.87	165^a^, 133, 104, 51	28	*Y* = 7.9722*x* − 0.0188	0.999	109.65	2.85	0.99	3.11	2.83
*(−)‐β*‐Caryophyllene **(C17)**	0.15–12	2.65	8.82	161,133,119,105,93^a^,77	15	*Y* = 2.7850*x* + 0.00066	0.999	108.93	2.88	1.11	3.95	3.32
Tridecane (ISTD) (**C18**)	—	—	—	184, 71^a^, 57, 43, 41	5	—	—	—	—	—	—	—

^a^ The ion was employed for quantitative detection.

**TABLE 3:** Analytical validation of the UPLC detection method.

Compound name	RT	Detection wavelength (nm)	Linear range (μg/mL)	LOD (μg/mL)	LOQ (μg/mL)	Calibration curve	*R* ^2^	Accuracy (%)	Accuracy (RSD%)	Precision (RSD %)	Repeatability (RSD%)	Stability (RSD%)
Synephrine (**C19**)	1.29	283	4.53–108.67	1.01	3.35	*Y* = 364.07*x* − 1089.4	0.9994	95.19	2.72	0.66	1.30	0.94
Protocatechuic acid (**C20**)	4.97	254	0.38–30.53	0.02	0.07	*Y* = 17,000*x* − 41.985	0.9999	101.39	2.26	1.45	1.81	0.94
Vanillic acid (**C21**)	7.66	254	1.34–53.78	0.03	0.09	*Y* = 17,117*x* − 2948.3	1.0000	100.48	2.92	1.54	1.31	1.13
Ferulic acid (**C22**)	10.73	330	0.02–1.44	0.0005	0.002	*Y* = 25,835*x* − 93.03	0.9998	101.31	2.50	2.44	2.88	1.13
Narirutin (**C23**)	12.10	283	1.89–189.3	0.07	0.22	*Y* = 8514.7*x* − 17,243	0.9991	99.52	2.19	1.06	2.07	3.20
Hesperidin (**C24**)	12.84	283	213.3–2133	0.01	0.04	*Y* = 4851.5*x* + 29,677	0.9999	102.56	1.70	0.87	2.86	0.47
Sinensetin (**C25**)	20.72	330	1.63–162.8	0.005	0.02	*Y* = 22,168*x* − 38,902	0.9992	103.52	1.59	1.06	1.01	0.60
Nobiletin (**C26**)	23.38	330	18.62–446.78	0.005	0.02	*Y* = 19,186*x* − 32,516	1.0000	97.67	3.06	2.25	1.01	0.48
Tangeretin (**C27**)	24.11	330	17.65–423.70	0.005	0.02	*Y* = 22,580*x* − 63,222	0.9999	98.19	3.72	2.03	1.04	0.38

The content of 17 volatile compounds was detected by the newly developed GC–MS method, and the results are detailed in Table 4. D‐Limonene, a prominent aromatic compound found in citrus fruits (Wen et al., 2022), exhibited the highest content in both XHCP‐0Y and XHCP‐3Y samples, ranging from 42.62 to 78.36 mg/g. Following closely was γ‐terpinene, known for enhancing aroma, with a content ranging from 1.93 to 13.28 mg/g. Despite being the stereoisomer of γ‐terpinene, α‐terpinene had significantly lower content, only 0.06 ~ 0.25 mg/g. Terpinolene, another isomer of γ‐terpinene, had a content range of 0.11 ~ 0.7 mg/g. The study also found relatively high content of α‐pinene, β‐pinene, and β‐myrcene, ranging from 0.38 to 1.55, 0.17 to 1.16, and 0.91 to 1.93 mg/g, respectively. Dimethyl anthranilate, a compound differentiating GCP and CP (Lv et al., 2020), was detected at 0.03 ~ 0.74 mg/g. Terpineol and (−)‐β‐caryophyllene followed with content ranging from 0.02 ~ 0.19 and 0.01 ~ 0.11 mg/g, respectively. However, seven other compounds, including linalool, citronellal, (+)‐4‐terpineol, DL‐carvone, (S)‐(−)‐perillaldehyde, thymol, and carvacrol, had content less than 0.1 mg/g, ranging from 0.02 ~ 0.07, 0.02 ~ 0.08, 0.01 ~ 0.03, 0.01 ~ 0.03, 0.02 ~ 0.07, 0.02 ~ 0.07, and 0.03 ~ 0.04 mg/g, respectively.

**TABLE 4:** The descriptor aroma and content (mg/g) of the 17 volatile compounds in XHCP‐0Y and XHCP‐3Y samples.

Compounds	Descriptor aroma	Content (mg/g)					
		0Y‐1	0Y‐2	0Y‐3	3Y‐1	3Y‐2	3Y‐3
*α*‐Pinene **(C1)**	Fresh, camphor and earthy woody odor	1.53 ± 0.03	1.55 ± 0.04	1.23 ± 0.02	0.38 ± 0.01	0.6 ± 0.05	0.96 ± 0.03
*β*‐Pinene **(C2)**	Woody‐green pine‐like smell	1.12 ± 0.04	1.16 ± 0.04	0.92 ± 0.03	0.17 ± 0.01	0.29 ± 0.05	0.75 ± 0.04
*β*‐Myrcene **(C3)**	Ethereal, resinous, soapy, spicy odor (pleasant)	1.93 ± 0.03	1.93 ± 0.03	1.77 ± 0.03	0.91 ± 0.02	1.32 ± 0.03	0.92 ± 0.02
*α*‐Terpinene **(C4)**	Woody, lemon‐flavored	0.25 ± 0.04	0.24 ± 0.04	0.19 ± 0.03	0.06 ± 0.03	0.08 ± 0.02	0.13 ± 0.04
*D*‐Limonene **(C5)**	Citrus, ethereal, fruity odor	78.36 ± 0.01	77.09 ± 0.01	73.88 ± 0.01	44.44 ± 0.01	61.75 ± 0.01	42.62 ± 0.01
*γ*‐Terpinene **(C6)**	Pine‐like smell, woody, lemon‐flavored	13.18 ± 0.04	13.28 ± 0.04	10.97 ± 0.03	1.93 ± 0.01	3.54 ± 0.01	8.32 ± 0.01
Terpinolene **(C7)**	Citrus flavor	0.7 ± 0.03	0.67 ± 0.05	0.54 ± 0.05	0.11 ± 0.01	0.18 ± 0.01	0.35 ± 0.05
Linalool **(C8)**	Aniseed, citrus, floral, terpene (pleasant scent)	0.06 ± 0.01	0.05 ± 0.02	0.07 ± 0.01	0.03 ± 0.01	0.04 ± 0.01	0.02 ± 0.02
Citronellal **(C9)**	Japanese pepper tree, floral, lemon scent	0.07 ± 0.03	0.08 ± 0.01	0.06 ± 0.01	0.02 ± 0.01	0.02 ± 0.01	0.02 ± 0.03
*(+)‐4*‐terpineol **(C10)**	Camphoraceous, earthy, musty odor (pleasant)	0.04 ± 0.01	0.04 ± 0.02	0.04 ± 0.01	0.04 ± 0.01	0.04 ± 0.01	0.04 ± 0.02
Terpineol **(C11)**	Pleasant odor	0.14 ± 0.01	0.13 ± 0.02	0.19 ± 0.01	0.02 ± 0.01	0.02 ± 0.01	0.12 ± 0.02
*DL*‐carvone **(C12)**	Minty	0.02 ± 0.02	0.02 ± 0.01	0.03 ± 0.03	0.01 ± 0.01	0.01 ± 0.02	0.01 ± 0.02
*(S)‐(−)*‐Perillaldehyde **(C13)**	Minty, herbal	0.05 ± 0.02	0.05 ± 0.01	0.07 ± 0.03	0.02 ± 0.01	0.02 ± 0.01	0.02 ± 0.02
Thymol **(C14)**	Herbal, pleasant aromatic odor	0.04 ± 0.02	0.07 ± 0.03	0.04 ± 0.03	0.02 ± 0.02	0.02 ± 0.01	0.03 ± 0.02
Carvacrol **(C15)**	Spicy	0.04 ± 0.03	0.04 ± 0.04	0.04 ± 0.05	0.03 ± 0.01	0.04 ± 0.01	0.04 ± 0.03
Dimethyl anthranilate **(C16)**	Floral	0.61 ± 0.05	0.74 ± 0.04	0.71 ± 0.04	0.03 ± 0.03	0.04 ± 0.01	0.05 ± 0.04
*(−)‐β*‐caryophyllene **(C17)**	Spicy	0.09 ± 0.04	0.11 ± 0.01	0.09 ± 0.05	0.01 ± 0.01	0.01 ± 0.01	0.08 ± 0.03

**FIGURE 3:**
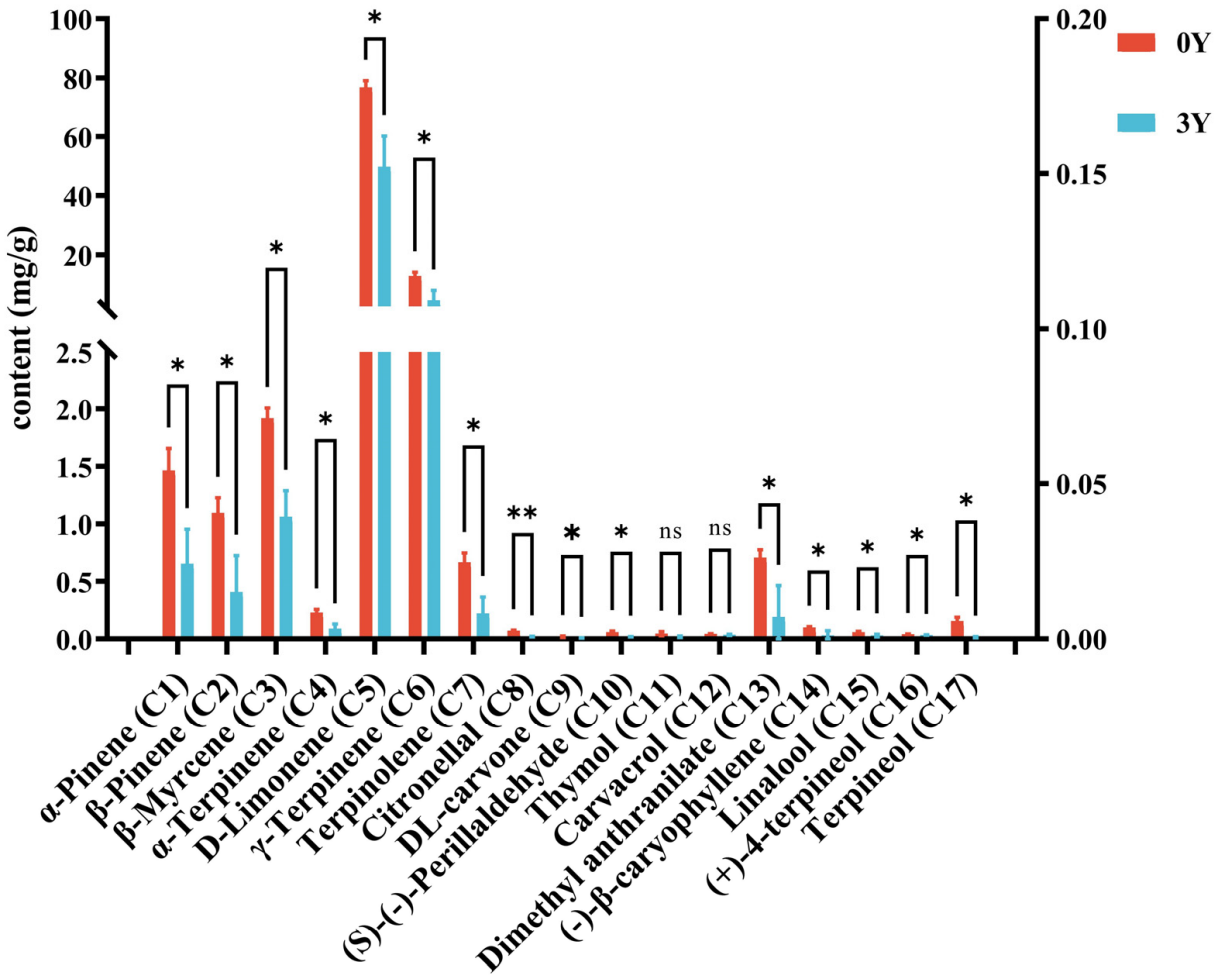
Content comparison (*n* = 3) of 17 volatile compounds in XHCP samples by GC–MS detection. The red histograms (**0Y**) represent the XHCP‐0Y sample, and the blue histograms (**3Y**) represent XHCP‐0Y sample. The ns (*p* ≥ .05) means no significance, and the five‐pointed star above the histogram indicates statistical significance at the level of .05 or .01 (*p ≤ .05, **p ≤ .01).

To facilitate a comparison of the content variations in 17 volatile compounds between XHCP‐0Y and XHCP‐3Y samples, column charts were generated using the average content of these compounds across three batches of XHCP. The results depicted in Figure 3 indicate a decline in the content of 17 volatile compounds from XHCP‐0Y to XHCP‐3Y samples. Apart from thymol and carvacrol, significant decreases were observed in the other 15 compounds, with citronellal showing particularly high significance. The aroma and flavor of XHCP are intricately linked to the content and proportion of volatile compounds, as detailed in Table 4. Compounds such as α‐terpinene, D‐limonene, γ‐terpinene, terpinolene, linalool, and citronellal contribute citrus or lemon notes and can be perceived as pungent at higher concentrations. Conversely, β‐myrcene, linalool, and (+)‐4‐terpineol are associated with pleasant aromas (Yi et al., 2015). It is widely accepted that high‐quality XHCP should exude a pleasant, pure, and mild scent. The varying levels of these volatile compounds caused by aging process may contribute to the unique aroma and flavor profile of XHCP. These findings align with Yi's research on PCR, which demonstrated that an initial high concentration of limonene resulted in an unpleasant smell in fresh PCR, but as its content decreased over time, the scent became more mellow (Yi et al., 2015). Additionally, traditional Chinese medicine theory posits a relationship between volatile compounds and dryness (Jin et al., 2014). The concentration of volatile compounds in freshly harvested CP is initially high, leading to strong dryness. However, the decrease in volatile compounds in CP during aging process may reduce the risk of adverse effects on patients with Yin deficiency (Yu et al., 2018). Despite this, the essential oil derived from CP exhibits a range of beneficial biological activities such as antioxidant, anti‐inflammatory, analgesic, antimicrobial, anticancer, expelling phlegm, and cough suppression (Singh et al., 2021). Further investigation is necessary to understand how the decrease in volatile compounds impacts the effectiveness of XHCP. The method developed in this study for detecting volatile compounds can serve as a basis for future investigations into the bioactivity of these compounds in XHCP during the aging process. In general, the decline in volatile compound content in XHCP could enhance its flavor and reduce the risk of dryness‐related side effects, potentially contributing to the overall improvement in quality during the aging process.

### 3.2 Analysis and detection of nonvolatile compounds by UPLC

**FIGURE 4:**
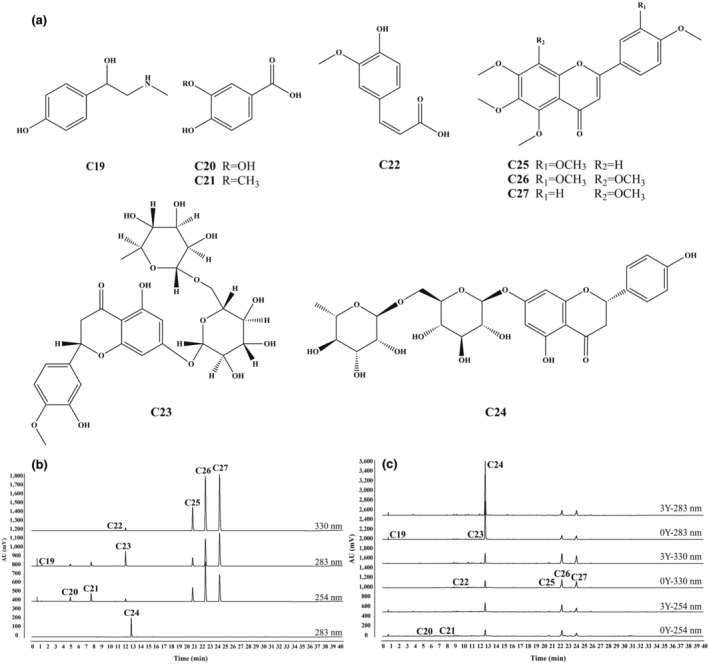
The chemical structure of nine nonvolatile compounds (**C19**–**C27**) (a), UPLC detection chromatograms of nine chemical reference substances (CRS) solution (b) and XHCP sample solutions (c) at 254, 283, and 330 nm. 0Y refers to XHCP‐0Y samples, and 3Y refers to XHCP‐3Y samples. The compounds **C19** and **C23** are synephrine (**C19**) and narirutin (**C23**), respectively, which were detected at 283 nm. The compound **C24** is hesperidin, which was detected separately due to its high concentration requirement at 283 nm. The compounds **C20** and **C21**, are protocatechuic acid (**C20**) and vanillic acid (**C21**), respectively, which were detected at 254 nm. The compounds, **C22, C25, C26,** and **C27**, are ferulic acid (**C22**), sinensetin (**C25**), nobiletin (**C26**), and tangerine (**C27**), respectively, which were detected at 330 nm.

In addition to volatile compounds, CRP contains various nonvolatile bioactive compounds such as flavonoids, phenolic acids, and alkaloids (Yu et al., 2018). As a commonly used traditional Chinese medicine in China, the quality control for GCP in the Chinese Pharmacopeia focuses on detecting three flavonoids: hesperidin, nobiletin, and tangeretin. However, the quality control does not extend to other types of compounds like phenolic acids and alkaloids. While the detection of flavonoids and phenolic acids in CRP has been reported due to their testability, significance, and beneficial properties (Zhao et al., 2016), these components are typically analyzed separately using different chromatographic conditions in HPLC, which increases testing time and cost. Therefore, building upon previous research, we developed and validated a simultaneous determination method using UPLC for nine main nonvolatile compounds of XHCP. These compounds included one alkaloid (synephrine, **C19**), three phenolic acids (protocatechuic acid, vanillic acid, ferulic acid, **C20**–**C22**), and five flavonoids (narirutin, hesperidin, sinensetin, nobiletin, and tangeretin, **C23**–**C27**). The structure information of these nine compounds is visually represented in Figure 4a. Detection wavelengths for the compounds were carefully selected based on absorbance comparisons across full wavelengths, resulting in 254 nm for **C20–C21**, 283 nm for **C19**, **C23–C24**, and 330 nm for **C22, C25–C27**. The final elution gradient, flow rate, and column temperature were optimized to enhance the resolution of each compound. The chromatograms of nine CRS (Figure 4b), XHCP‐0Y sample, and XHCP‐3Y sample (Figure 4c) demonstrated the specificity and resolution of the newly developed method. Table 4 presents the results of the method validation conducted in accordance with Section 2.5. The LOQ and LOD were determined to be lower than 0.5 ng/mL, meeting the detection requirements. The *R* ^2^ for the linearities exceeded 0.999. The RSD% for precision and repeatability was below 3%. Furthermore, the stability of the sample solution was confirmed, as the RSD% remained under 4% over a 24‐h period. These findings indicate the method's strong performance. Subsequently, the content of nine nonvolatile compounds in XHCP‐0Y and XHCP‐3Y samples was detected by the UPLC method, as shown in Table 5. Hesperidin had the highest content in both samples, ranging from 45.863 to 80.389 mg/g. Nobiletin, tangerine, and synephrine followed with content ranging from 4.062 to 5.358 mg/g, 2.87 to 3.833 mg/g, and 1.463 to 2.095 mg/g, respectively. The remaining flavonoids, narirutin and sinensetin, had content ranging from 0.452 to 1.159 mg/g and 0.483 to 0.583 mg/g. The content of the three phenolic acids, protocatechuic acid, vanillic acid, and ferulic acid, was found to be less than 0.5 mg/g.

**TABLE 5:** The content (mg/g) of the nine nonvolatile compounds in XHCP‐0Y and XHCP‐3Y samples.

Compounds	0Y‐1	0Y‐2	0Y‐3	3Y‐1	3Y‐2	3Y‐3
Synephrine (**C19**)	2.044 ± 0.11	2.092 ± 0.13	2.095 ± 0.1	1.463 ± 0.06	1.531 ± 0.05	1.521 ± 0.04
Protocatechuic acid **(C20)**	0.043 ± 0.03	0.043 ± 0.04	0.044 ± 0.02	0.069 ± 0.03	0.075 ± 0.05	0.074 ± 0.09
Vanillic acid **(C21)**	0.086 ± 0.02	0.085 ± 0.03	0.086 ± 0.01	0.104 ± 0.04	0.114 ± 0.07	0.114 ± 0.03
Ferulic acid **(C22)**	0.190 ± 0.05	0.186 ± 0.04	0.187 ± 0.07	0.417 ± 0.02	0.437 ± 0.04	0.43 ± 0.06
Narirutin **(C23)**	0.487 ± 0.07	0.453 ± 0.06	0.452 ± 0.02	1.018 ± 0.05	1.089 ± 0.08	1.159 ± 0.1
Hesperidin **(C24)**	50.679 ± 0.22	47.328 ± 0.19	45.863 ± 0.15	65.391 ± 0.18	75.967 ± 0.2	80.389 ± 0.21
Sinensetin **(C25)**	0.487 ± 0.03	0.483 ± 0.01	0.483 ± 0.05	0.558 ± 0.04	0.583 ± 0.06	0.57 ± 0.05
Nobiletin **(C26)**	4.074 ± 0.1	4.062 ± 0.11	4.078 ± 0.07	5.099 ± 0.04	5.358 ± 0.03	5.223 ± 0.05
Tangerine **(C27)**	2.878 ± 0.08	2.87 ± 0.07	2.885 ± 0.12	3.651 ± 0.09	3.833 ± 0.11	3.738 ± 0.13

**FIGURE 5:**
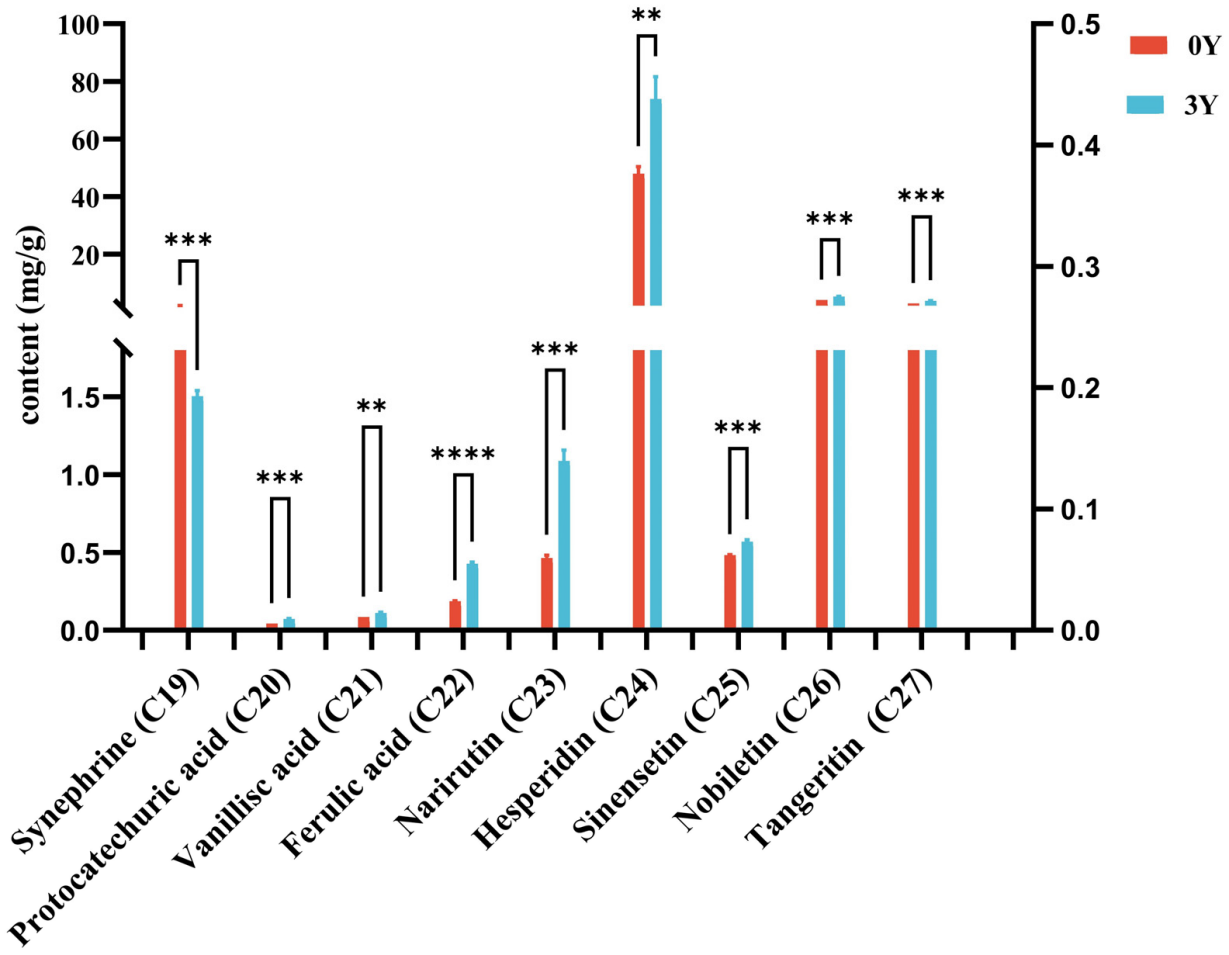
Content comparison (*n* = 3) of nine non‐volatile compounds in XHCP samples by UPLC detection. The red histograms (**0Y**) represent the XHCP‐0Y sample, and the blue histograms (**3Y**) represent XHCP‐0Y sample. The five‐pointed star above the histogram indicates the statistical significance at the level of .01, .001, or .0001. (**p ≤ .01, ***p ≤ .001, ****p ≤ .0001).

Figure 5 presents a comparison of the nine nonvolatile compounds in XHCP‐0Y and XHCP‐3Y samples. Previous studies have mainly focused on the variation of synephrine during the aging process in samples stored for 2, 3, or more years (Wei et al., 2013). This study is the first to report a significant decrease in synephrine content from XHCP‐0Y to XHCP‐3Y. Synephrine, recognized as the active component in plants and dietary supplements used for weight loss, is commonly found in plants of the Rutaceae family (Rossato et al., 2011). Although synephrine is generally considered to have few side effects and high safety, recent studies have raised concerns about its cardiovascular toxicity and adverse reactions associated with dietary supplements due to its structural resemblance to adrenergic agonists (Stohs et al., 2020). Consequently, further comprehensive investigations are warranted to elucidate the link between the decline in synephrine content during storage and its potential toxicological effects. The content of three phenolic acids (**C20–C22**) and five flavonoids (**C23**–**C27**) showed a significant increase during storage. Previous research has indicated that the main bioactivities of *Citrus* are attributed to phenolic acids and flavonoids, which are abundant compared to other components (Benavente‐García & Castillo, 2008; Ho & Kuo, 2014; Tao et al., 2022). The increase in the content of these compounds may account for the observed quality improvement in XHCP during the aging process, which linked this enhancement to heightened antioxidant activity and the inhibitory effects on glucose‐hydrolysis enzymes in CP and GCP (Bian et al., 2022; Zhang et al., 2014). The aging process of CP had been explored partly, noting changes in compounds attributed to self‐degradation or microbial presence. Volatile compounds are known to decrease in volatility under varying storage conditions of temperature and humidity. Microorganisms such as *Bacillus*, *Lactococcus*, and *Pseudomonas* have been identified as critical factors in the biotransformation of volatile oils, leading to an increase in phenolic and flavonoid content in CRP (Chen, 2017; He, 2019; Wang et al., 2015; Yang et al., 2021, 2022). Nevertheless, further research is required to elucidate the specific mechanisms by which microorganisms induce these transformations.

### 3.3 CV detection

The color of CP is known to deepen with age, becoming a key factor in differentiating CP stored for varying durations. Initially, freshly harvested XHCP appears orange or dark green, eventually transitioning to brown or dark brown over time. However, discrepancies in color perception among observers have been noted. To address this issue, a spectrophotometer was utilized in this study to analyze changes in the color values of XHCP‐0Y and XHCP‐3Y samples, focusing on the L*, a*, and b* parameters. The most significant change was observed in the L* value, decreasing from 62.11 to 43.07, indicating a darkening of the color. Changes in the a* value were relatively gradual, increasing from 5.73 to 11.19. While the b* value showed a slow decline from 32.31 to 27.79. These findings suggest that color changes, aside from lightness and darkness, were not very pronounced. However, the ∆E* value was 34.15 ± 0.97, indicating that the colors of the XHCP‐0Y and XHCP‐3Y samples belonged to two distinct color categories (Table 6). These observed color changes are attributed to the food browning phenomenon, a common occurrence during storage, particularly in fruits (Hamdan et al., 2022). Previous research by Zhu has indicated that the gradual depletion of β‐carotene and ascorbic acid serve as precursors, while polyphenols and reducing sugars are the primary contributors to GCP browning (Zhu et al., 2022). Nonetheless, it is imperative to investigate whether these color changes have any implications on the efficacy of CRP in the medical context.

**TABLE 6:** The color values of XHCP‐0Y and XHCP‐3Y samples.

Sample name	L*	a*	b*	∆E*
XHCP‐0Y	62.11 ± 1.72	5.73 ± 0.15	32.31 ± 0.59	—
XHCP‐3Y	43.07 ± 0.02	11.19 ± 0.03	27.79 ± 0.03	34.15 ± 0.97

### 3.4 Detection of TF and AA

**FIGURE 6:**
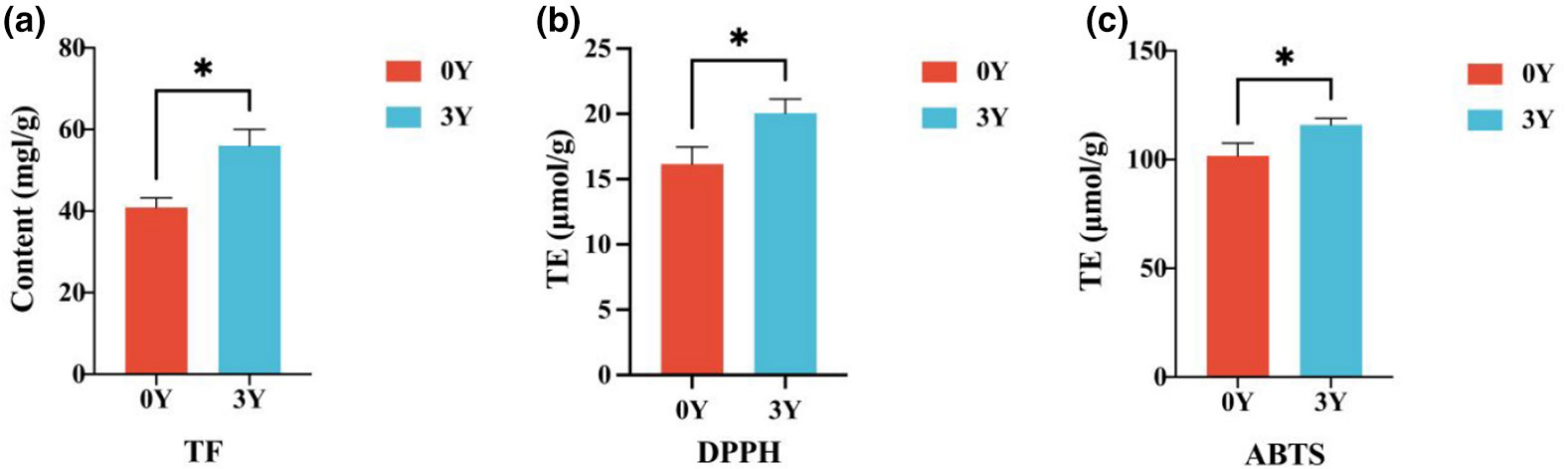
Comparison of total flavonoids (a), antioxidant activity by DPPH (b) and ABTS (c) in XHCP. The red histograms (0Y) represent the XHCP‐0Y sample, and the blue histograms (3Y) represent XHCP‐0Y sample. The five‐pointed star above the histogram indicates the statistical significance at the level of .05. (*p ≤ .05).

Flavonoids, such as hesperidin, nobiletin, and tangerine, are the primary active components in CP, sharing a common nuclear structure and detectable at the same wavelength (Yi et al., 2009). A calibration curve for TF detection was established using varying concentrations of hesperidin CRS solutions. The TF content in XHCP‐0Y sample was measured at 40.89 ± 2.34 mg/g, while in XHCP‐3Y sample, it was 56.06 ± 3.98 mg/g (Figure 6a). These results indicate a significant increase in TF content over storage time, aligning with findings in other cultivated varieties like CP and GCP with different storage years (Wang et al., 2016).

The antioxidant activity of XHCP was evaluated using DPPH and ABTS assays. The DPPH value for XHCP‐0Y sample was 16.15 ± 1.31 μmol TE/g, and for XHCP‐3Y sample, it was 20.05 ± 1.09 μmol TE/g (Figure 6b). Similarly, the ABTS value for XHCP‐0Y samples was 101.68 ± 5.84 μmol TE/g, and for XHCP‐3Y samples, it was 115.98 ± 2.99 μmol TE/g (Figure 6c). These results clearly show that the XHCP‐3Y sample displays significantly higher antioxidant activity compared to XHCP‐0Y sample. This increase in activity may be attributed to increased levels of flavonoids and phenolic acid. Flavonoids have been reported to scavenge reactive oxygen species, counteract lipid oxidation in vitro, boost the body's antioxidant enzyme activity, and diminish peroxide formation in vivo. Phenolic acids showcase potent antioxidant properties through dehydrogenation of hydroxyl groups and ortho‐substitution impact on a benzene ring (Bursal et al., 2021; Zou et al., 2016).

## 4 CONCLUSION

CRP is one of the six traditional Chinese medicines that need an aging process. Currently, the standard for traditional Chinese medicine in Guangdong Province mandates that Xinhui Chenpi, a type of CRP, must be aged for a minimum of 3 years. However, limited research exists on the rationale behind this aging requirement. This study aimed to compare freshly harvested XHCP (XHCP‐0Y) with XHCP aged for 3 years (XHCP‐3Y). Initially, the volatile compounds of XHCP were analyzed using GC–MS, resulting in the identification of 38 common volatile compounds. Subsequently, 17 differential volatile compounds were selected for quantitatively detection through PCA, OPLS‐DA, VIP analysis, and the availability of chemical reference standards. Additionally, based on literature research, nine nonvolatile compounds, including an alkaloid (synephrine), three phenolic acids (protocatechuic acid, vanillic acid, and ferulic acid), and five flavonoids (narirutin, hesperidin, sinensetin, nobiletin, and tangeretin), were selected for quantitative detection. Finally, two simple and rapid methods were first developed for the detection of the 17 volatile and nine nonvolatile compounds in XHCP with varying storage durations using GC–MS and UPLC. Validation of these newly developed methods confirmed their accuracy and efficiency. Chemical components and quality analysis revealed a significant decrease in the content of 15 volatile compounds and synephrine, along with an increase in the content of three phenolic acids, five flavonoids, total flavonoids, antioxidant activity, and color values during the storage of XHCP. These findings suggest an enhancement in the quality of XHCP over time. Additionally, our study offers a new data foundation for selecting XHCP that has been stored for more than 3 years, aligning with the regulations of the Standard for Traditional Chinese Medicine in Guangdong Province. Nevertheless, further research is needed to fully understand the internal mechanisms and the implications of these changes on in vivo activity during the aging process.

## AUTHOR CONTRIBUTIONS

**JunJie Shi:** Conceptualization (equal); data curation (lead); formal analysis (lead); methodology (equal); resources (equal); software (equal); validation (equal); visualization (equal); writing – original draft (lead); writing – review and editing (equal). **Lihua Peng:** Conceptualization (equal); data curation (supporting); investigation (equal); methodology (supporting); software (supporting); validation (supporting); visualization (supporting); writing – review and editing (equal). **Weixuan Chen:** Conceptualization (equal); data curation (supporting); investigation (equal); methodology (equal); visualization (equal); writing – review and editing (equal). **Weilin Qiao:** Funding acquisition (equal); investigation (equal); project administration (equal); supervision (equal); writing – review and editing (equal). **Kui Wang:** Project administration (equal); supervision (equal); writing – review and editing (equal). **Yueyang Xue:** Formal analysis (supporting); resources (equal); validation (equal). **Jinle Cheng:** Conceptualization (equal); funding acquisition (lead); writing – review and editing (equal).

## CONFLICT OF INTEREST STATEMENT

The authors declare that they have no conflict of interest.

## Supporting information


Figures S1–S2

## Data Availability

The data that support the findings of this study are available from the corresponding author upon reasonable request.
