# Supplementary material for: Evaluation of chemical components and quality in Xinhui Chenpi (Citrus reticulata ‘Chachi’) with two different storage times by GC–MS and UPLC
Source: Food Sci Nutr. 2024 Apr 10;12(7):5036–51. doi: 10.1002/fsn3.4154 (PMC11266906; doi:10.1002/fsn3.4154)
Supplement: Supplementary file 1 — Figures S1–S2 [file FSN3-12-5036-s001.docx]

**Supplementary figure**


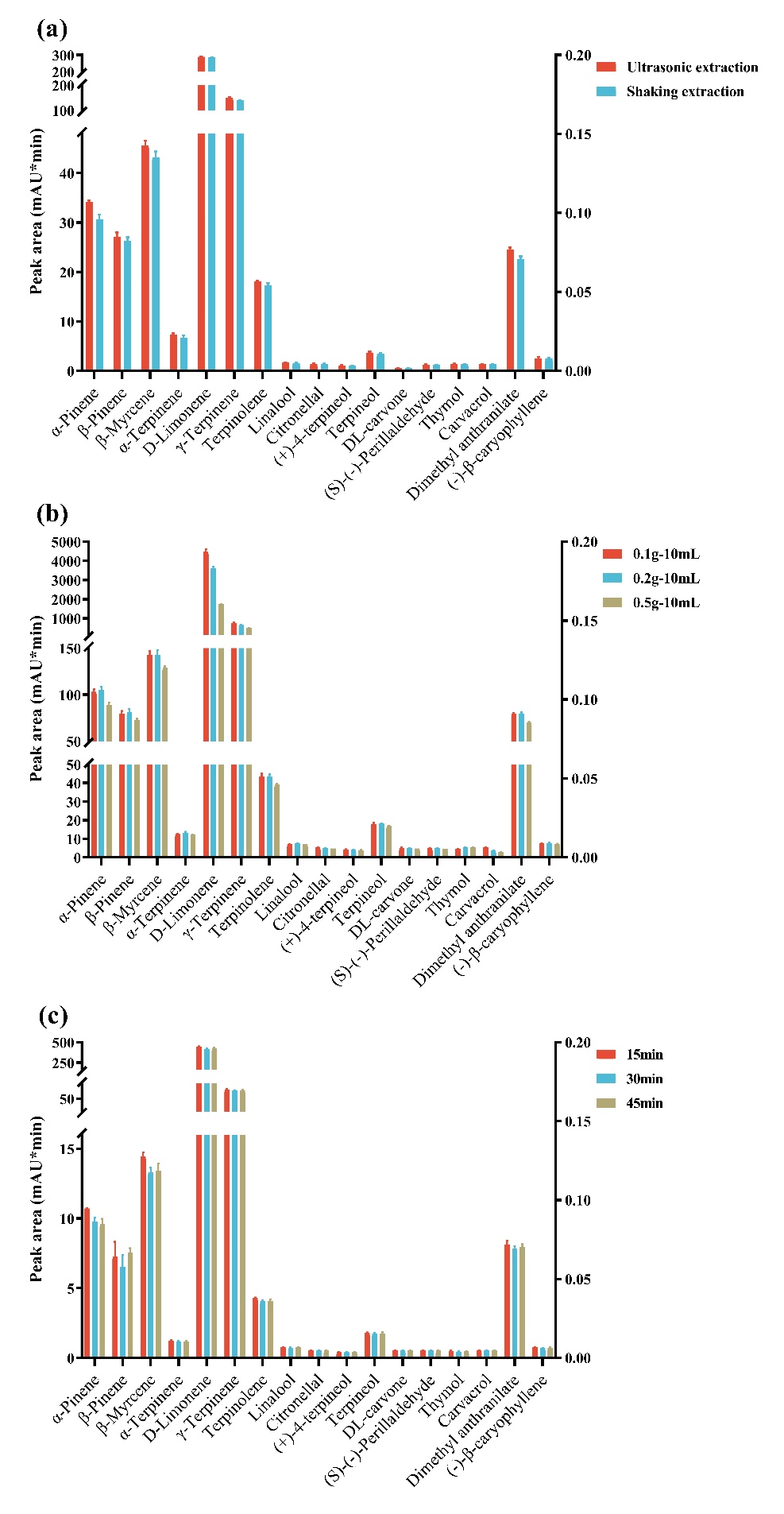


**Fig. S1**. Effect of the extraction method (a), feed-to-liquid ratio (b), and extraction time (c), on the peak area of the seventeen volatile compounds


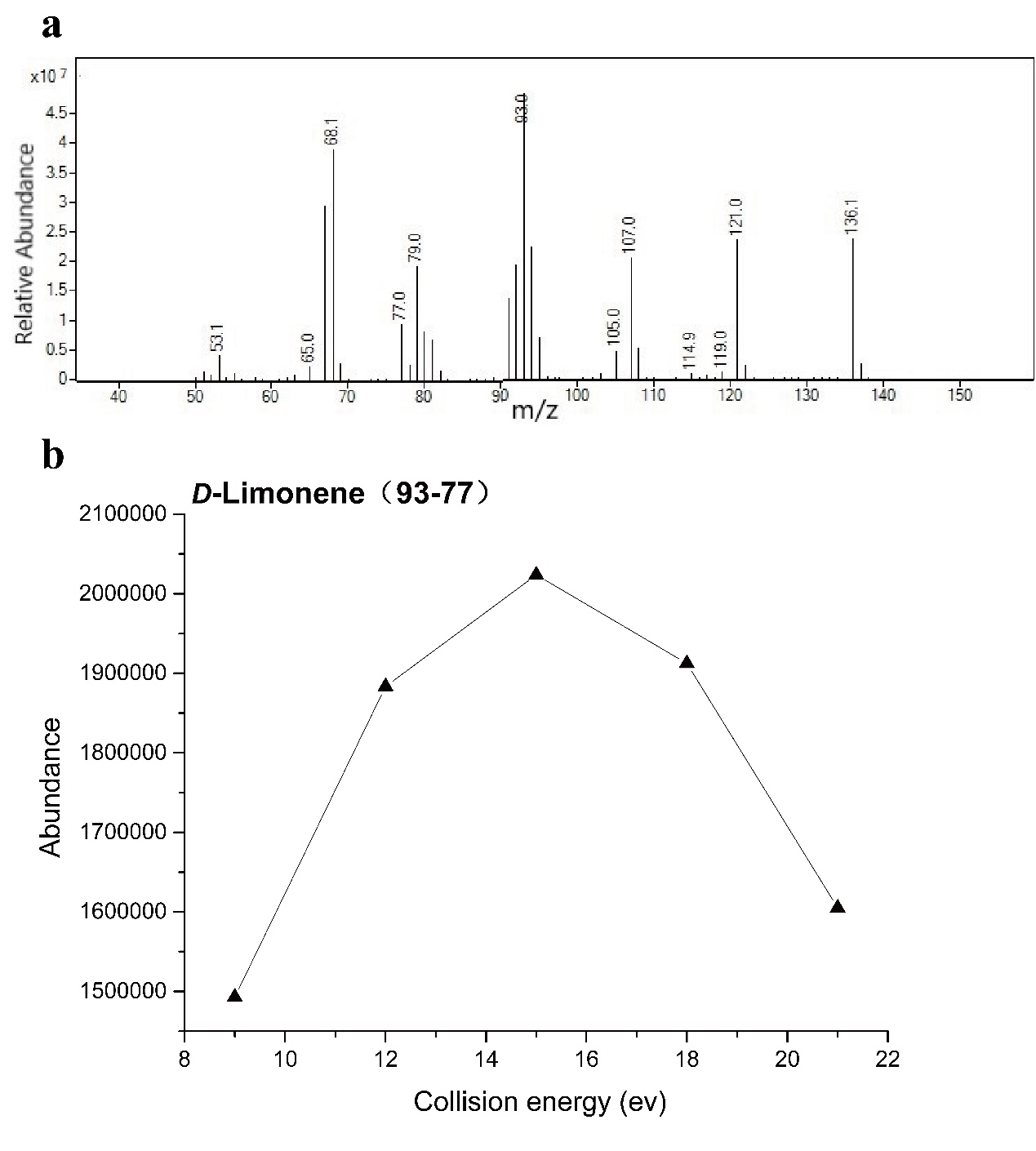


**Fig. S2**. The characteristic ions (a) and collision energy (b) for *D*-limonene
